# Geographical and seasonal variations of soil microbiomes and metabolomes in the core production area of Jiang-flavor Baijiu: a multi-omics characterization

**DOI:** 10.3389/fmicb.2026.1816391

**Published:** 2026-04-27

**Authors:** Qiong Li, Zhe Li, Guang Zeng, Maomao Zhang, Fujun Wang, Pengyun Chen, Songxian Yan

**Affiliations:** 1 Moutai Institute, Renhuai, Guizhou, China; 2 School of Food and Liquor Engineering, Sichuan University of Science and Engineering, Zigong, China; 3 Guizhou Xijiu Co., Ltd., Xishui, Guizhou, China; 4 College of Agronomy and Biotechnology, China Agricultural University, Beijing, China; 5 Guizhou Engineering Research Center for Comprehensive Utilization of Distillers' Grains, Renhuai, Guizhou, China

**Keywords:** flavor formation, geographical location, Jiang-flavor Baijiu, metabolome, multi-omics integration, seasonal variation, soil microbiome

## Abstract

The unique flavor of Chinese Jiang-flavor Baijiu is hypothesized to be influenced by the regional environment. However, the specific contributions of soil microbiomes and metabolomes remain poorly characterized. This study systematically analyzed soil phenotypes, microbiomes, and metabolomes at five sampling sites near the Moutai production area across spring and autumn. Using high-throughput sequencing, untargeted metabolomics, and multi-omics integration, we explored the impacts of geographical location and seasonal changes. Results revealed significant differences in soil microbial biomass, dominant taxa, and differential metabolites among sites. Region-specific marker microorganisms and metabolic pathways were identified. Seasonal variations, particularly in Region A, strongly affected metabolite profiles. Multi-omics correlation analysis revealed that *Ascomycota* abundance was positively associated with flavor precursor metabolites including phenylpropanoids, organosulfur compounds, and nucleosides, which are known precursors of aromatic compounds found in Baijiu. This study characterizes the distinct ecological profiles of soils in the core production area, providing a foundational dataset for understanding the regional environmental context of Jiang-flavor Baijiu. These findings offer insights for protecting the regional ecological integrity associated with production areas.

## 1 Introduction

With a time-honored history, Chinese baijiu is a clear distilled beverage crafted via intricate fermentation procedures. It joins brandy, whiskey, vodka, rum, and gin as one of the six most widely favored distilled liquors across the globe (Xu et al., 2017; Tu et al., 2022). The term “baijiu” translates literally to “white alcohol” and is classified as an alcoholic drink. Regarded as China’s national liquor, baijiu prides itself on a long-standing heritage and distinctive brewing methods, holding a significant role in the economic structure of China’s food industry. In 2020, the sales revenue of Chinese baijiu hit 90.33 billion US dollars, while its annual sales volume surpassed 10.7 billion liters (Tu et al., 2022).

Chinese Baijiu can be classified into different types based on its flavor, such as Jiangxiang (Jiang-flavor), Nongxiang (Strong-flavor), Qingxiang (Light-flavor), and Miangxiang (Rice-flavor) (Zheng and Han, 2016). The production of Jiang-flavor Baijiu mainly consists of four key steps: Qu-making (starter-making), alcohol fermentation, solid-state distillation, and aging. During brewing, starchy raw materials like sorghum are mixed with unique high-temperature Qu (starter). The mixture undergoes natural fermentation in an open environment, adopting a two-stage alcohol fermentation process involving “heap fermentation” and “pit fermentation”—with no direct or indirect addition of edible alcohol, nor color, aroma, or taste substances that are not produced by its own fermentation (Jin et al., 2017). Moutai Liquor is the most renowned type of Jiang-flavor Baijiu, featuring prominent mellow taste and a long-lasting aftertaste, and 528 compounds have been identified in this kind of Jiang-flavor Baijiu (Zhu et al., 2007).

Microorganisms act as the critical bridge connecting the environment to Baijiu’s flavor, and this linkage is part of the interactive system formed between the environment, microorganisms, and Baijiu’s flavor during the liquor’s fermentation (Wang, 2022). During the fermentation process, microorganisms generate volatile substances such as esters, alcohols, acids, aldehydes, nitrogen-containing compounds, sulfur-containing compounds, and terpenes through their metabolic activities (Zheng and Han, 2016; Jin et al., 2017; Wu et al., 2024). A major factor behind the notable quality differences in Baijiu from different production sites lies in the unique conditions of Baijiu brewing: it depends on an open environment for inoculation and involves a fermentation process that is highly complex (Wang et al., 2015). This concept parallels the terroir effect in wine production, where soil microbiomes and regional environmental factors contribute distinctly to product characteristics (Gilbert et al., 2014).

Numerous studies have indicated that microorganisms thriving in unique ecological environments take part in the fermentation of Chinese Baijiu, facilitating the formation of various flavor components during the Baijiu-brewing process (Calasso et al., 2016). For instance, when microorganisms of the genus *Bacillus* are added to the fermentative microbial community, the contents of sulfur-containing compounds, pyrazine compounds, and acids in Chinese Baijiu increase significantly. Additionally, the initial fungal diversity exerts a positive impact on the succession of the fungal community during fermentation, which suggests that initial fungal diversity plays a crucial role in promoting the formation of flavor compounds in Chinese Baijiu (Shen et al., 2020; Ji et al., 2022). Long-term Baijiu production activities, coupled with the region’s suitable climatic conditions, help maintain a relatively stable microbial community structure in the surrounding environment of Baijiu-producing areas, such as soil (Wu et al., 2024).

Despite growing recognition of the environment-microbe-flavor nexus, several knowledge gaps remain. Specifically, which specific microorganisms affect the metabolites in Jiang-flavor Baijiu, and how these microorganisms interact with the metabolites, remain unelucidated. Furthermore, the relative contributions of geographical location versus seasonal variation to soil microbiome and metabolome profiles in baijiu-producing regions have not been systematically compared.

In this study, we first analyzed the soil phenotypes at different geographical locations near the Jiang-flavor Baijiu production area. Subsequently, we comprehensively employed high-throughput sequencing and untargeted metabolomics techniques, combined with multi-omics integrative analysis, to systematically investigate the effects of geographical location and seasonal variations on the soil metabolome and microbiome. This research aims to systematically characterize the soil phenotypes, microbiomes, and metabolomes across different geographical locations and seasons within the core production area. We aim to identify region-specific marker microorganisms and metabolites, and to explore potential associations between the soil environment and the microbial metabolic potential relevant to Baijiu fermentation.

## 2 Materials and methods

### 2.1 Sample collection

Soil samples were collected from five sampling sites within the same geographical area near a Jiang-flavor Baijiu production town in Maotai, Renhuai City, Guizhou Province, China. Site A is located on a small hill on the side of the valley in the town where a certain Jiang-flavor Baijiu distillery is located; the soil here is sandy soil, covered with vegetation on the surface, with coordinates of 27°50′6″N, 106°21′38″E and an altitude of 604 m. Site B is situated in the middle of the valley on the side of the town; it has loamy soil, with vegetation covering the surface, and its coordinates are 27°50′33″N, 106°21′1″E, at an altitude of 567 m. Site C lies beside the Chishui River; the soil type is sandy soil, with surface vegetation, and it is located at 27°50′29″N, 106°21′15″E, with an altitude of 398 m. Site D is located in the middle of the opposite side of the valley in the town; the soil here is loam, covered with plants on the surface, and its coordinates are 27°50′36″N, 106°21′11″E, at an altitude of 569 m. Site E is located on the summit of the mountain on the opposite side of the valley in the town; it has sandy-gravel soil, with a small amount of weeds on the surface, and is located at 27°51′14″N, 106°21′16″E, with an altitude of 758 m.

Sampling was conducted on two dates representing different seasons: May 2, 2022 (spring) and December 11, 2022 (autumn). On May 2, 2022 (spring sampling), Maotai Town, Renhuai City experienced cloudy conditions with a northwest wind (level 1), temperatures ranging from 16 to 22 °C, relative humidity of 85%, and no precipitation. On December 11, 2022 (autumn sampling), the same site had cloudy weather with a south wind (level 1), temperatures between 9 and 11 °C, relative humidity of 85%, and zero rainfall. Samples were collected at the same sampling sites in each season, and three independent replicate samples were taken at each site during each sampling period to ensure the representativeness and repeatability of the sampling.

Prior to soil collection, the surface litter layer (1–2 cm) was removed to eliminate fallen leaves, weeds, stones, and root debris. Subsequently, soil samples from the 0–20 cm depth were collected using sterile stainless steel shovels. The collected soil was homogenized using the quartering method and reduced to approximately 50 g per sample. Samples were immediately flash-frozen in dry ice and transported to the laboratory for subsequent sequencing and metabolomic analyses. During the sampling process, environmental information such as GPS coordinates and soil physicochemical parameters was recorded for each site.

### 2.2 ITS1 amplicon sequencing and data processing

Total genomic DNA was extracted from soil samples using the TGuide S96 Magnetic Soil/Feces Genome DNA Extraction Kit (TIANGEN, DP812) according to the manufacturer’s instructions. For fungal community analysis, the ITS1 region was amplified using the primer pair ITS1F (5′-CTTGGTCATTTAGAGGAAGTAA-3′) and ITS2 (5′-GCTGCGTTCTTCATCGATGC-3′). PCR products were purified using VAHTS DNA Clean Beads (Vazyme, N411-03) to remove residual primers and short fragments. The purified amplicon libraries were sequenced on the Illumina NovaSeq 6,000 platform using a 2 × 150 bp paired-end strategy. The purified libraries were then sequenced on the Illumina NovaSeq 6,000 platform (Illumina, San Diego, USA) using a 2 × 150 bp paired-end configuration. The sequencing data have been deposited in the NCBI database under BioProject accession number SUB16072715.

The raw sequences obtained from sequencing were filtered using Trimmomatic v0.33, and the primer sequences were removed using cutadapt 1.9.1 (Martin, 2011; Bolger et al., 2014). Paired-end reads were merged using USEARCH v10, followed by quality filtering and length filtering based on the expected length distribution of the ITS1 region (Edgar, 2010).

For quality control, the effective read counts and Q30 values were calculated to ensure data reliability. The processed sequences were clustered into ASVs (Amplicon Sequence Variants) using the DATA2 algorithm within the QIIME2 (version 2020.6) pipeline to correct sequencing errors and distinguish biological sequences from noise (Licata et al., 2025). Based on the R package “phyloseq,” *α*-diversity (including Chao1, Shannon index, etc.) and *β*-diversity (PCoA) indices were calculated (Watson et al., 2013). Additionally, the R package “microeco” was used for LEfSe analysis to evaluate inter-group differences (Liu et al., 2021).

### 2.3 Metabolomics data acquisition and data processing

Soil samples were subjected to metabolite extraction using a methanol/water solution. The extraction process involved grinding and ultrasonic treatment. After standing and centrifugation, the supernatant was collected for vacuum drying, followed by reconstitution with an appropriate amount of extraction solution. The resulting samples were used for liquid chromatography-mass spectrometry (LC–MS) analysis, utilizing the Waters Acquity I-Class PLUS ultra-high-performance liquid chromatography system coupled with the Waters Xevo G2-XS QTOF high-resolution mass spectrometer.

Raw data acquired by MassLynx V4.2 were processed using Progenesis QI software for peak extraction, peak alignment, and other data processing operations. Metabolite identification was performed based on the online METLIN database integrated in Progenesis QI software, as well as public databases (KEGG, HMDB), combined with theoretical fragment identification. The mass deviation was controlled within 100 ppm for parent ions and 50 ppm for fragment ions.

Orthogonal partial least squares discriminant analysis (OPLS-DA) was conducted on the normalized metabolite data using the R package “ropls” to screen for metabolites with significant changes between different groups (VIP > 1) (Thévenot et al., 2015). Additionally, the R package “clusterProfiler” was used for KEGG enrichment analysis to investigate metabolic pathways (Wu et al., 2021).

### 2.4 Multi-omics integrative analysis

The correlation between metabolite abundances and microbial community abundances was analyzed by calculating Spearman correlation coefficients, and the results were visualized using a heatmap generated with the R package “pheatmap” (Hu, 2021). In addition, the “circosPlot” function from the R package “mixOmics” was used to display the associations between soil phenotypes and geographically related differential metabolites and differential microorganisms (Schneidman et al., 2017).

## 3 Results

### 3.1 Differences in soil phenotypes across different geographical locations

**Figure 1:**
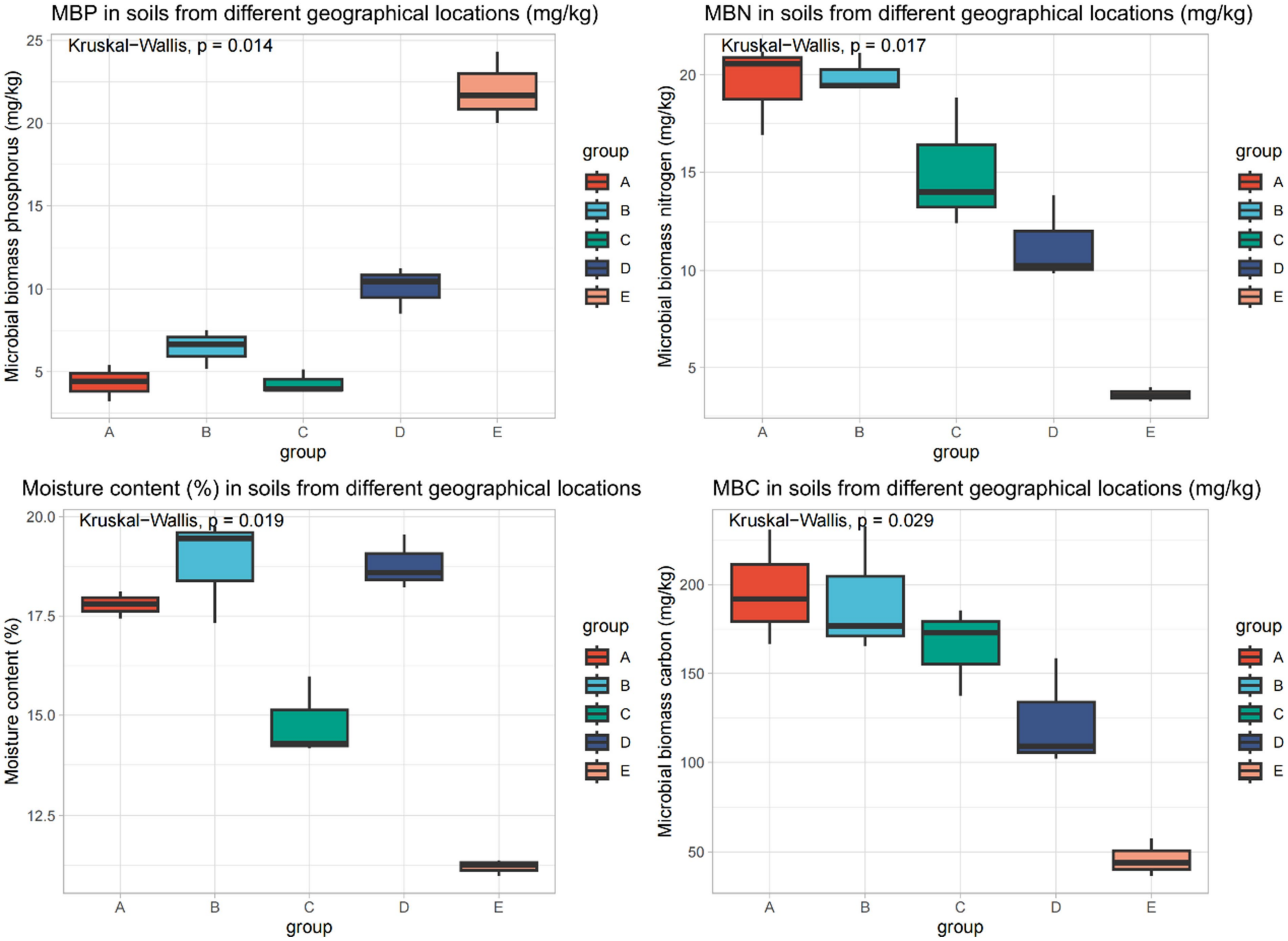
Differences in spring soil phenotypes across different geographical locations.

To explore the differences in soil phenotypes across different geographical locations, the microbial biomass carbon (MBC), microbial biomass phosphorus (MBP), microbial biomass nitrogen (MBN), and moisture content of spring soils in five regions were determined. The Kruskal-Wallis results showed significant differences among the different groups (MBP: *p* = 0.014, MBN: *p* = 0.017, moisture content: *p* = 0.019, MBC: *p* = 0.029): Regions A and B had relatively high levels of soil MBC, MBN, and moisture content, but low MBP; Region C had high soil MBC, yet low MBP, MBN, and moisture content; Region D had high moisture content, while its MBC, MBP, and MBN were low; Region E had high MBP, but low MBC, MBN, and moisture content (Figure 1; Supplementary Table S2).

### 3.2 Impact of geographical location on soil microbiome

**Figure 2:**
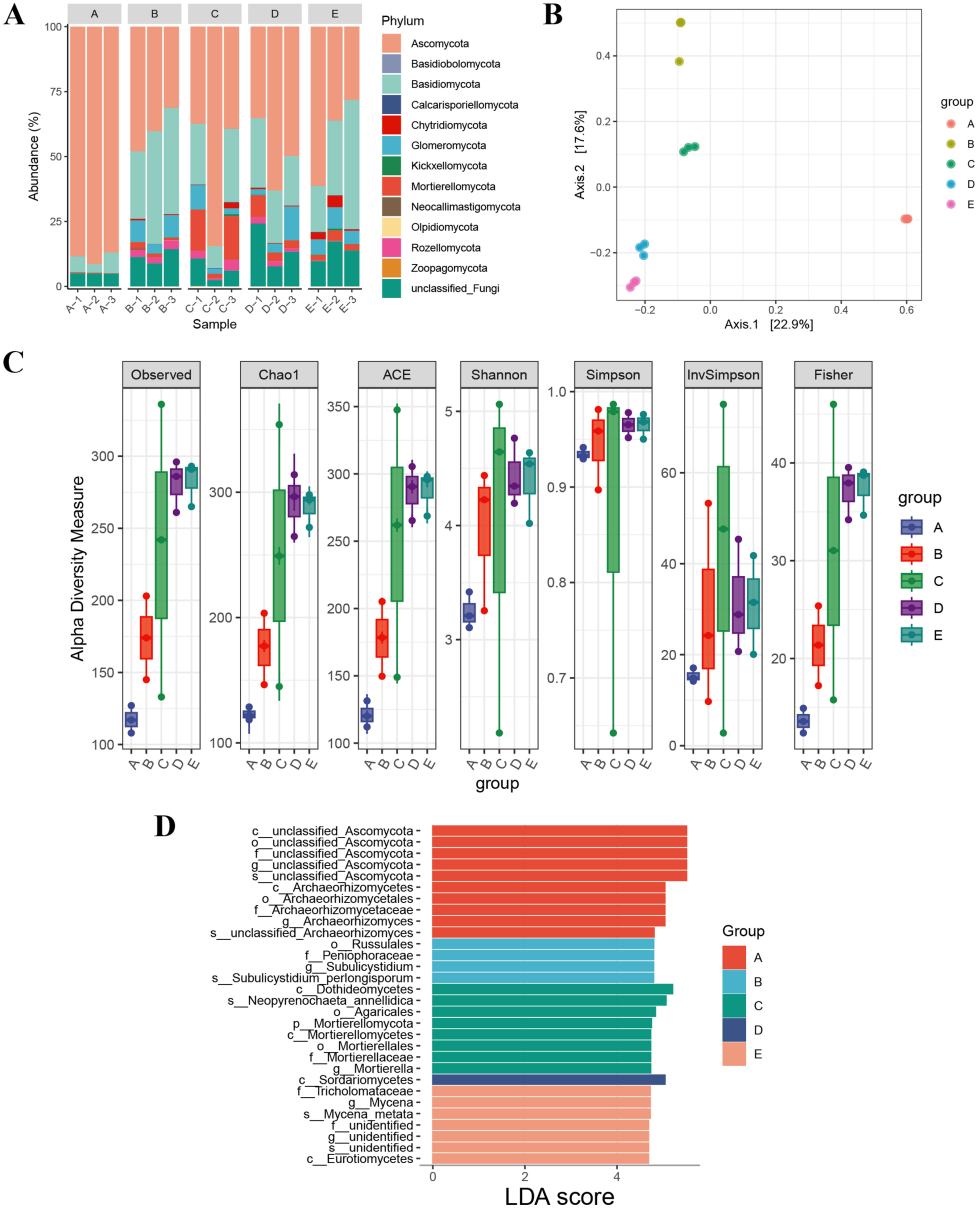
Impact of geographical location on soil fungal community structure: **(A)** Relative abundance of dominant fungal phyla across five sampling sites; **(B)** *β*-diversity of fungal communities; **(C)** Alpha diversity of fungal communities; **(D)** Linear Discriminant Analysis Effect Size (LEfSe) analysis of fungal communities.

To investigate the differences in soil microbiomes across different geographical locations, ITS sequencing analysis was conducted (Supplementary Table S1). The microbial community abundance map of the 5 sample groups is shown in Figure 2A. At the phylum level, a total of 12 phyla were identified, with the main dominant phyla including Ascomycota and Basidiomycota. Region A had the highest relative abundance of Ascomycota, while Region C had the highest relative abundance of Mortierellomycota among the five regions. Principal Coordinates Analysis (PCoA) results indicated that the samples from the 5 locations were clearly separated (Figure 2B). Alpha diversity analysis results showed that the species diversity in Regions D and E was relatively high, while that in Regions A and B was relatively low (Figure 2C). LEfSe analysis results revealed that the abundance of Archaeorhizomycetes was significant in Region A; the abundances of Russulales and Peniophoraceae were significant in Region B; the abundance of Dothideomycetes was significant in Region C; the abundance of Sordariomycetes was significant in Region D; and the abundance of Tricholomataceae was significant in Region E (Figure 2D).

### 3.3 Impact of geographical location on soil metabolites

**Figure 3:**
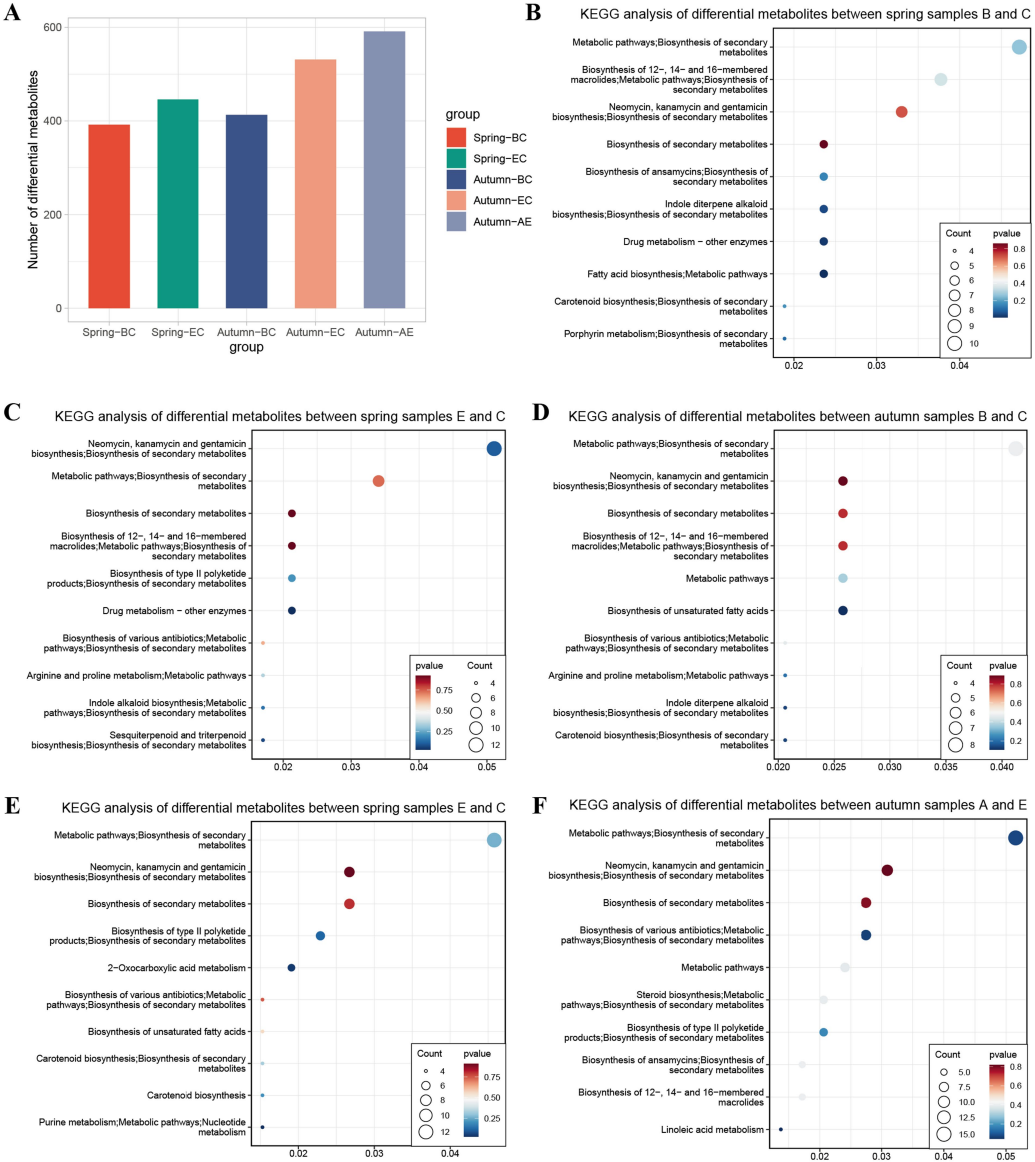
Geographical variation in soil metabolites and their functional enrichment. **(A)** The number of differential metabolites between pairwise comparisons of five sampling sites in spring and autumn **(B–F)**. KEGG enrichment bubble plots of differential metabolites for key pairwise site comparisons.

To study the differences in soil metabolomes across different geographical locations, soil metabolites were collected from the 5 geographical locations in both spring and autumn. Pairwise comparisons were made among the five locations, and Orthogonal Projections to Latent Structures-Discriminant Analysis (OPLS-DA) was used to screen for differential metabolites (Figure 3A). The results showed that there were differential metabolites between Regions B and C, and Regions E and C in spring, as well as between Regions B and C, Regions E and C, and Regions A and E in autumn. Moreover, the number of differential metabolites between Regions A and E in autumn was the largest. Kyoto Encyclopedia of Genes and Genomes (KEGG) enrichment analysis was performed on the differential metabolites (Figures 3B–F; Supplementary Table S3). It was found that the differential metabolites between Regions B and C in spring were mainly associated with pathways such as the biosynthesis of secondary metabolites and fatty acid biosynthesis; the differential metabolites between Regions E and C in spring were mainly related to pathways including the biosynthesis of neomycin, kanamycin, gentamicin, and secondary metabolites; the differential metabolites between Regions B and C in autumn were mainly linked to the biosynthesis of unsaturated fatty acids; the differential metabolites between Regions E and C in autumn were mainly associated with pathways such as 2-oxobutanoate metabolism; and the differential metabolites between Regions A and E in autumn were mainly related to pathways including the biosynthesis of various antibiotics, metabolic pathways, and biosynthesis of secondary metabolites (Figures 3B–F; Supplementary Table S3).

### 3.4 Impact of seasonal changes on soil metabolites

**Figure 4:**
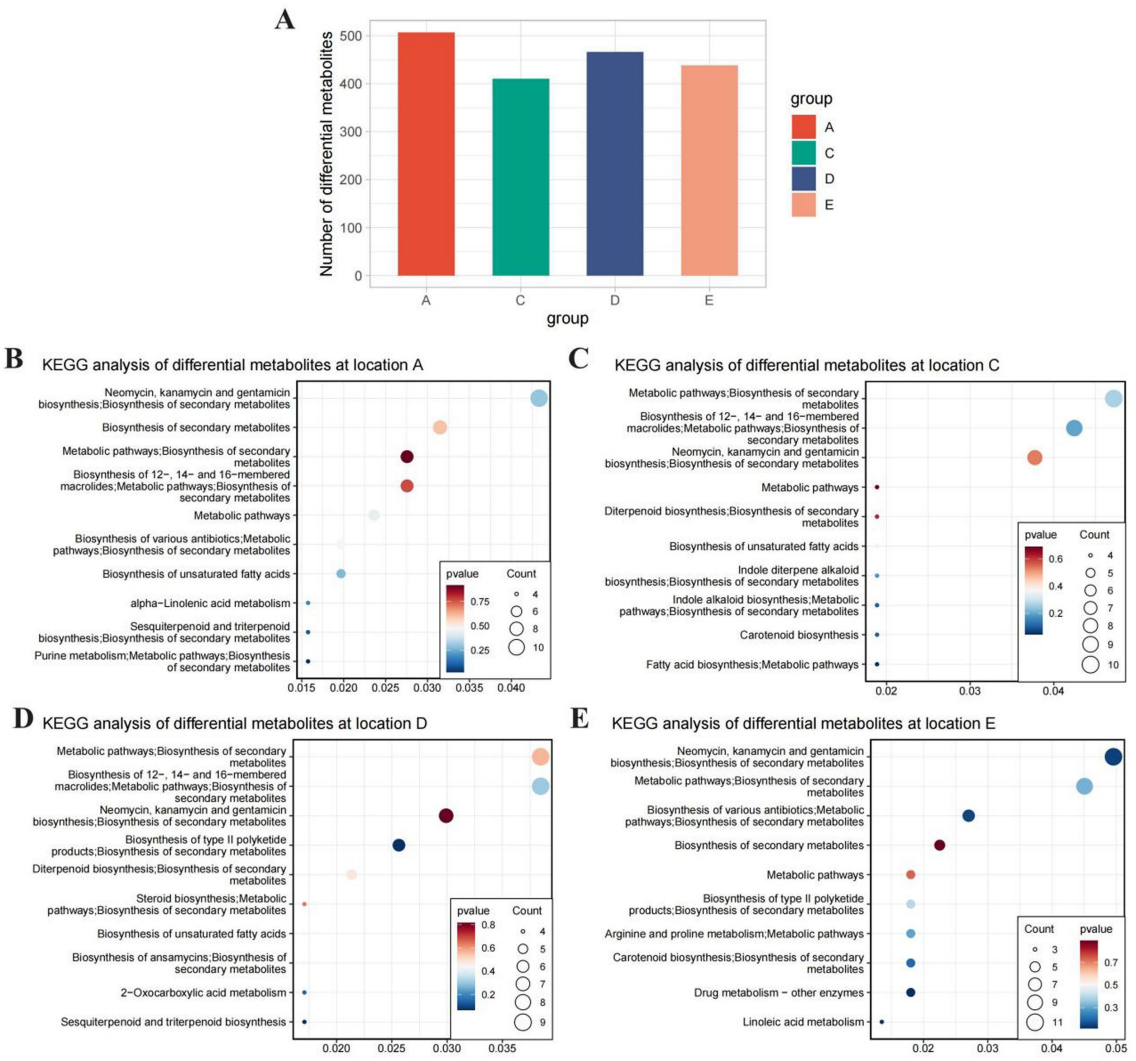
Seasonal variation in soil metabolites and their functional enrichment: **(A)** The number of differential metabolites and **(B–E)** KEGG enrichment bubble plots of seasonal differential metabolites for each site.

In addition to studying the impact of geographical location on soil metabolites, soil samples were collected in spring and autumn for metabolomic analysis. OPLS-DA was performed on the normalized metabolite data (Figure 4A). The OPLS-DA results showed that there were significant differences in soil metabolites between spring and autumn in Regions A, C, D, and E, and Region A had the largest number of differential metabolites. Enrichment analysis of the differential metabolites revealed that the differential metabolites between spring and autumn in Region A were mainly associated with pathways such as the biosynthesis of neomycin, kanamycin, gentamicin, and secondary metabolites; the differential metabolites in Region C were mainly related to pathways including the biosynthesis of macrolide antibiotics, metabolic pathways, and biosynthesis of secondary metabolites; the differential metabolites in Region D were mainly linked to pathways such as the biosynthesis of type II polyketide products and biosynthesis of secondary metabolites; and the differential metabolites between spring and autumn in Region E were associated with the biosynthesis of some antibiotics (Figures 4B–E; Supplementary Table S4).

### 3.5 Correlation analysis between metabolites and microbial communities

**Figure 5:**
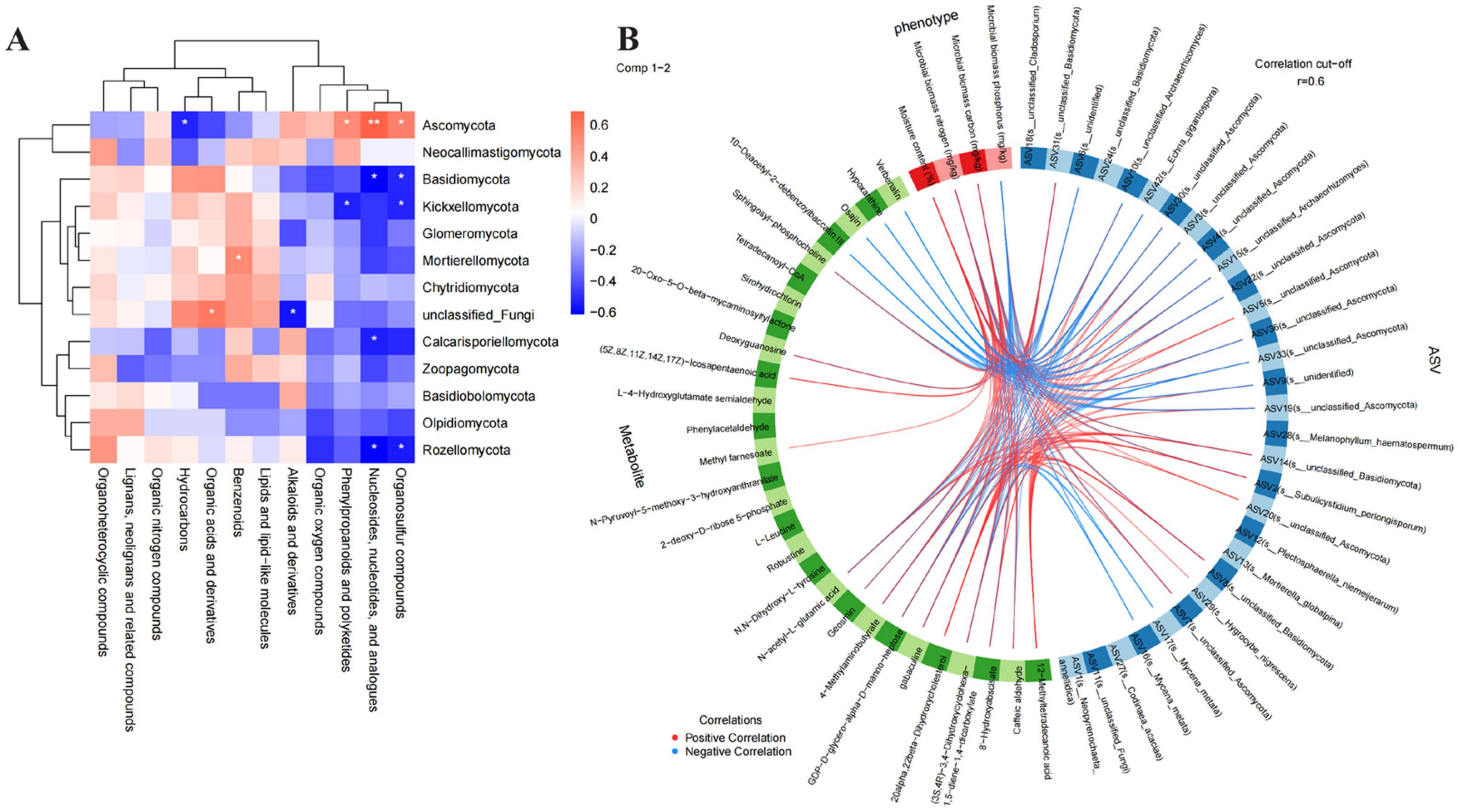
Multi-omics integrative analysis of soil fungi, metabolites, and phenotypes: **(A)** Spearman correlations between fungal phyla and metabolite classes; **(B)** associations among soil phenotypes (red segment), metabolites (green segment), and fungi (blue segment).

To study the correlation between metabolite counts and microbial community abundance, the Spearman correlation coefficient was calculated (Figure 5A). The results showed that Ascomycota was significantly positively correlated with nucleosides, nucleotides, and analogues, organosulfur compounds, and phenylpropanoids and polyketides; Basidiomycota and Rozellomycota were significantly negatively correlated with organosulfur compounds, as well as nucleosides, nucleotides, and analogues. However, it should be noted that these correlations do not imply causal relationships, and the observed associations may be influenced by other environmental factors.

In addition, the potential associations between soil phenotypes and some location-related differential metabolites and differential microorganisms were also explored (Figure 5B). The results showed that MBN was positively correlated with substances such as N-acetyl-L-glutamic acid and negatively correlated with microorganisms such as Mycena metata; MBC was positively correlated with substances such as gabaculine and negatively correlated with microorganisms such as certain Ascomycota species; soil moisture content was positively correlated with substances such as methyl farnesoate and microorganisms such as certain *Ascomycota* species; MBP was negatively correlated with substances such as deoxyguanosine and microorganisms such as certain *Basidiomycota* species (Figure 5B). These findings suggest potential interactions between soil physicochemical properties, microbial communities, and metabolite profiles, though further experimental validation would be required to establish causal mechanisms.

## 4 Discussion

### 4.1 Different environments provide abundant microbial growth conditions

This study systematically analyzed soil phenotypes, microbiomes, and metabolomes across five geographical locations near the Jiang-flavor Baijiu production area, and explored the impacts of geographical location and seasonal changes, as well as the correlations among multi-omics data. These sites have distinct niche functions: Region A (cemetery beside a new factory of the Jiang-flavor Baijiu distillery) is a control for non-production area disturbance, retaining indigenous microbial pools; Region B (above the Starter-making Workshop) is a core production interaction zone, serving as a “source-sink” for brewing microorganisms; Region C (beside Chishui River) is a riparian material convergence zone; Region D (below Jingjiu Distillery) verifies industrial cluster effects; Region E (under viewing platform cableway) is a natural background control with minimal brewing interference.

The results revealed significant differences in soil phenotypes among different regions. Regions A and B exhibited high levels of microbial biomass carbon (MBC), microbial biomass nitrogen (MBN), and moisture content but low microbial biomass phosphorus (MBP), while Region E showed the opposite pattern with high MBP and low MBC, MBN, and moisture content (Figure 1; Supplementary Table S2). This aligns with the previous findings, and emphasized that unique geological and soil properties in Baijiu-producing regions shape soil microbial habitats and further drive variations in soil biological indicators like microbial biomass (Wu et al., 2024).

Notably, Region C had high MBC but low MBP, MBN, and moisture content (Supplementary Table S2). This could be attributed to the specific hydrological conditions of the riverside environment. Although the Chishui River basin provides high-quality water sources rich in minerals, the periodic fluctuation of river water levels might lead to rapid leaching of soil moisture and soluble nutrients, resulting in low soil moisture content and limited availability of nitrogen and phosphorus nutrients. In contrast, Region D had high moisture content but low microbial biomass, which may be related to the potential impact of long-term distillery activities on the soil environment (Tu et al., 2022; Wu et al., 2024).

### 4.2 Responses of soil microbiome to geographical location and ecological drivers

The ITS sequencing results indicated that *Ascomycota* and *Basidiomycota* were the dominant fungal phyla across all regions, which is consistent with the microbial community characteristics of high-temperature Daqu and fermented grains in Jiang-flavor Baijiu production systems (Figure 2A) (Tu et al., 2022).

Specifically, Region A had the highest relative abundance of *Ascomycota*, while Region C had the highest relative abundance of *Mortierellomycota* (Figure 2A). This regional specificity in microbial community composition may be driven by differences in environmental factors. For example, the higher altitude of Region A (604 m) and its sandy soil texture with vegetation cover might create a microclimate suitable for the growth of Ascomycota, whereas the warm and humid riverside environment of Region C (altitude 398 m) could favor the proliferation of *Mortierellomycota* (Wang, 2022). Alpha diversity analysis showed that Regions D and E had higher species diversity than Regions A and B (Figure 2C). This pattern may be linked to the combined effects of soil texture and human disturbance. Region E had sandy-gravel soil with only a small amount of weeds, and its relatively low level of human disturbance might allow for the maintenance of a more diverse microbial community. In contrast, Regions A and B, which are close to production facilities, may experience selective pressure from human activities, leading to a reduction in microbial diversity (Tu et al., 2022; Wu et al., 2024).

LEfSe analysis identified region-specific marker microorganisms, such as the high abundance of *Archaeorhizomycetes* in Region A and *Tricholomataceae* in Region E (Figure 2D). These microorganisms may play crucial roles in local soil nutrient cycling and metabolite synthesis. For example, *Archaeorhizomycetes* are widely involved in the decomposition of soil organic matter, and their high abundance in Region A may contribute to the accumulation of MBC (Kerjean et al., 2000; Tu et al., 2022). *Tricholomataceae*, as a group of ectomycorrhizal fungi, can form symbiotic relationships with plants to promote nutrient absorption, which may explain the relatively high MBP in Region E (Zhu et al., 2022). And PCoA analysis further confirmed the clear separation of microbial communities among different regions, indicating that geographical location is a key factor driving the differentiation of soil microbial communities, which is consistent with the conclusion of the previous study (Figure 2B) (Wu et al., 2024). Factors such as soil texture, altitude, moisture content, and nutrient availability jointly shape the structure of soil microbial communities, and these differences in microbial communities may further affect the metabolic potential of the soil ecosystem.

### 4.3 Variations in soil metabolites driven by geographical location and seasons

OPLS-DA analysis revealed significant differences in soil metabolites among different geographical locations and between seasons. In terms of geographical location, the pairwise comparisons showed that the number of differential metabolites between Regions A and E in autumn was the highest (Figure 3A; Supplementary Table S3). This could be due to the large differences in environmental conditions between the two regions, which may lead to significant variations in microbial metabolic pathways. For example, the KEGG enrichment results showed that the differential metabolites between Regions A and E in autumn were mainly involved in the biosynthesis of various antibiotics and secondary metabolites. This is similar to the previous finding of microorganisms in unique ecological environments promote the formation of specific flavor components through secondary metabolic pathways (Tu et al., 2022). The high abundance of *Tricholomataceae* in Region E and Ascomycota in Region A may be the key microbial drivers of these metabolic pathway differences.

In terms of seasonal changes, Region A had the largest number of differential metabolites between spring and autumn, and these metabolites were mainly involved in the biosynthesis of neomycin, kanamycin, gentamicin, and secondary metabolites (Figure 3F; Supplementary Table S3). This suggests that the soil ecosystem of Region A is more sensitive to seasonal environmental changes. For example, the significant fluctuations in temperature and precipitation between spring and autumn in the subtropical monsoon climate of the Guizhou region may alter the activity of soil microorganisms, thereby regulating the synthesis and degradation of secondary metabolites (Wu et al., 2024). In contrast, the seasonal differences in metabolites in Region C were mainly related to the biosynthesis of macrolide antibiotics, which may be due to the buffering effect of the riverside environment on temperature and moisture, leading to relatively stable microbial metabolic activities (Figure 4C; Supplementary Table S4) (Tan et al., 2014).

It is worth noting that the differential metabolites between spring and autumn in multiple regions were involved in the biosynthesis of antibiotics. This may be a soil microbial adaptation strategy to cope with seasonal changes in biotic and abiotic stresses. For example, in autumn, the decrease in temperature and the increase in plant litter may increase competition among microorganisms, and the synthesis of antibiotics can help dominant microorganisms inhibit the growth of competitors (Xu et al., 2017; Wu et al., 2024).

### 4.4 Multi-omics correlations and their implications for baijiu fermentation

The Spearman correlation analysis showed that *Ascomycota* was significantly positively correlated with nucleosides, nucleotides, organosulfur compounds, and phenylpropanoids/polyketides, while *Basidiomycota* and *Rozellomycota* were significantly negatively correlated with these metabolites (Figure 5A). This indicates that the composition of the fungal community is associated with the accumulation of specific metabolites in the soil environment. For example, the presence of *Ascomycota* correlates with the abundance of phenylpropanoids and nucleosides, which are potential precursors for fermentation (Tu et al., 2022; Wu et al., 2024). In contrast, *Basidiomycota* may compete with *Ascomycota* for nutrients, thereby inhibiting the production of these metabolites. Notably, several of the identified fungal genera, such as *Saccharomyces*, *Aspergillus*, and *Penicillium*, are known in other fermentation contexts to possess enzymatic capabilities for converting precursors into aromatic compounds (Li et al., 2023; Dong et al., 2025). The detection of phenylpropanoids in the soil suggests a potential environmental reservoir of such compounds, although their direct transfer to the fermentation process requires further validation. Similarly, the detection of organosulfur compounds in the soil highlights a potential environmental source of sulfur-containing metabolites. While these compounds can be transformed into volatile sulfur compounds in fermentation systems, the direct link between the soil reservoir and the final Baijiu aroma profile remains to be established (Li et al., 2023). However, it should be emphasized that these correlations represent potential associations rather than direct causal relationships, and the actual conversion of soil metabolites into Baijiu flavor components would require microorganisms to be transferred from soil to the fermentation environment through multiple pathways (Tu et al., 2022; Wu et al., 2024).

The association analysis between soil phenotypes and differential metabolites/microorganisms further revealed the potential relationships of microbial-metabolite interactions regulating soil functions. For instance, microbial biomass nitrogen (MBN) was positively correlated with N-acetyl-L-glutamic acid and negatively correlated with *Mycena metata* (Figure 5B). This suggests that *M. metata* may inhibit the synthesis of amino acid metabolites by competing for nitrogen sources, thereby affecting the accumulation of MBN. Additionally, soil moisture content was positively correlated with methyl farnesoate and certain *Ascomycota* species. Terpenoids are important volatile flavor precursors of Baijiu, and this correlation implies that the soil moisture environment may regulate the synthesis of terpenoid metabolites through influencing the growth of *Ascomycota*, which in turn may affect the flavor formation of Jiang-flavor Baijiu (Xu et al., 2017; Wu et al., 2024).

Notably, microbial biomass phosphorus (MBP) was negatively correlated with deoxyguanosine and certain *Basidiomycota* species. This may be because *Basidiomycota* can secrete phosphatases to decompose organic phosphorus in the soil, increasing the availability of inorganic phosphorus. However, the consumption of phosphorus by *Basidiomycota* may reduce the phosphorus content available for other microorganisms, thereby limiting the synthesis of phosphorus-containing metabolites such as deoxyguanosine and ultimately leading to a decrease in MBP. Furthermore, the impact of industrial human activities on soil microbial communities has been well-documented. Anthropogenic activities can significantly alter soil physicochemical properties and microbial community structure, as demonstrated in various ecosystems including freshwater sediments and forest soils (Zhou et al., 2023). Long-term exposure to industrial activities such as distillery operations may lead to selective pressure on soil microorganisms, favoring taxa that are tolerant to environmental stressors such as elevated ethanol concentrations, organic acid accumulation, and fluctuating pH levels. These anthropogenic disturbances can result in reduced microbial diversity and shifts in community composition, as observed in Regions A and B compared to the less disturbed Region E (Figure 2C). Similar patterns have been reported in agricultural systems where human management practices significantly influenced soil microbial community diversity and function (Li et al., 2023; Zhou et al., 2023).

## 5 Conclusion

This study presents a comprehensive multi-omics profiling of the soil environment in the core Jiang-flavor Baijiu production area. We reveal that geographical location and seasonal changes are key drivers shaping the distinct soil microbiomes and metabolomes. These findings characterize the unique ecological context of this region, providing essential baseline data for future studies on the environmental factors potentially associated with Baijiu production. Therefore, protecting the integrity of this ecosystem is crucial for the sustainable development of the Jiang-flavor Baijiu industry.

## Data Availability

The sequencing data have been deposited in the NCBI database under BioProject accession number PRJNA1440252.

## Supplementary material

SUPPLEMENTARY TABLE S1 Summary of sequencing data quality control and filtering statistics for all samples.

SUPPLEMENTARY TABLE S2 Determination results of soil microbial biomass and moisture content at different sampling sites.

SUPPLEMENTARY TABLE S3 KEGG enrichment analysis results of differential metabolic pathways in soils between sampling sites across seasons.

SUPPLEMENTARY TABLE S4 TKEGG enrichment analysis results of characteristic metabolic pathways in soils of each sampling site.
